# GT-Mamba: a Topology-Aware Graph-State space model for robust and interpretable epigenetic age prediction

**DOI:** 10.1093/bioinformatics/btag401

**Published:** 2026-06-16

**Authors:** Han Wang, Hui Wang, Yanting Tong, Yuanyuan Liu, Qu Jing, Guan Ning Lin, Li Zhang

**Affiliations:** School of Information Science and Technology, Institute of Computational Biology, Northeast Normal University, Changchun 130117, China; School of Biomedical Engineering Excellence, China Pharmaceutical University, Nanjing 211198, China; School of Information Science and Technology, Institute of Computational Biology, Northeast Normal University, Changchun 130117, China; School of Information Science and Technology, Institute of Computational Biology, Northeast Normal University, Changchun 130117, China; School of Information Science and Technology, Institute of Computational Biology, Northeast Normal University, Changchun 130117, China; College of Computer Science and Technology, Jilin University, Changchun 130012, China; College of Computer Science and Engineering, Changchun University of Technology, Changchun 130051, China; College of Computer Science and Technology, Jilin University, Changchun 130012, China

## Abstract

**Motivation:**

Current epigenetic clocks face a trade-off between predictive accuracy and biological interpretability, often relying on dataset-specific correction to generalize across cohorts. We propose GT-Mamba, a novel architecture that integrates a Structure-Aware Graph Transformer with the Mamba state space model. This design captures CpG topological correlations and genome-wide long-range dependencies.

**Results:**

GT-Mamba demonstrates strong out-of-the-box robustness across heterogeneous independent validation cohorts, achieving a weighted average MAE of 4.43 years. Notably, it effectively generalizes to EPIC 850k arrays despite partial feature missingness, and maintains consistent performance across homologous age distribution shifts (MAE 2.94 years in a young cohort). Ablation studies confirm that graph topology contributes to improved robustness against noise. Mechanistic analysis suggests that the model captures methylation patterns associated with both developmental and functional processes.

**Availability:**

Source code and pre-trained models are freely available at https://github.com/NENUBioCompute/GT-Mamba and archived on Zenodo (DOI: 10.5281/zenodo.19703155).

## 1 Introduction

Genomic instability and epigenetic alterations are hallmarks of the aging process (López-Otín *et al.* 2023), with DNA methylation (DNAm) serving as a widely used biomarker for biological age estimation. While pioneering linear clocks such as Horvath (Horvath 2013) and Hannum (Hannum *et al.* 2013) established the field, they rely on independence assumptions that may not fully capture the complex, non-linear interactions within gene regulatory networks. Furthermore, prediction accuracy in whole blood is frequently confounded by cellular heterogeneity, where shifts in immune cell composition can obscure intrinsic intracellular aging signals (Bell *et al.* 2019). These challenges highlight the need for models that better capture both local interactions and global dependencies, enabling more robust identification of intrinsic aging signals.

To address these challenges, we propose *GT-Mamba*, a hierarchical framework that integrates biologically informed feature selection with graph deep learning. At the feature level, we introduce a *Mechanism-Anchored Residual Ensemble* strategy, which emphasizes biologically relevant CpG sites while incorporating complementary data-driven features to improve coverage. At the modeling level, GT-Mamba employs a “Local Topology–Global Sequence” architecture. A *Structure-Aware Graph Transformer* captures non-linear local interactions (Dwivedi and Bresson 2020), while a *Mamba* module leverages its input-dependent selective state space mechanism to dynamically filter biological noise and adaptively model distributed and heterogeneous epigenetic signals (Gu and Dao 2023). This design enables the joint modeling of local structural fidelity and global systemic context.

Extensive validation on an independent cross-platform meta-dataset (*N* = 1892, including a large-scale EPIC 850k cohort) demonstrates strong generalization capability. Evaluated in a strict out-of-the-box setting without post-hoc calibration, GT-Mamba achieves high predictive accuracy in high-quality cohorts and maintains robust performance under age distribution shifts (e.g. young cohorts). Notably, it remains effective when transferred to EPIC arrays despite partial feature missingness, highlighting its robustness under cross-platform variability. Moreover, biological correlation analysis suggests that the model mitigates confounding from cellular composition and captures intrinsic aging-related methylation signals. Overall, GT-Mamba provides a unified framework that balances predictive performance and biological interpretability, with potential applications in aging research and biomarker development.

## 2 Materials and methods

### 2.1 Data collection and preprocessing

We constructed a meta-dataset comprising 15 whole-blood cohorts (*N* = 7158, aged 2–101) from GEO (Barrett *et al.* 2013), partitioned into a development set (10 cohorts) and an independent external validation set (5 cohorts, *N* = 1892, including the large-scale EPIC 850k cohort GSE132203; Table S1, available as supplementary data at *Bioinformatics* online).

Raw data were processed using a hybrid R/Python pipeline. After filtering low-quality samples (missing rate >50% or missing age), approximately 380,000 CpG sites shared across the Illumina 450k arrays were retained. Missing values were imputed using the array-aware methyLImp2 algorithm (Di Lena *et al.* 2019), followed by BMIQ normalization to correct probe design bias. To preserve biological heterogeneity and reflect cross-cohort variability, no explicit batch correction was applied across cohorts.

### 2.2 Dual-driven feature selection strategy

#### 2.2.1 Data-driven screening

To isolate intrinsic aging signals from cellular heterogeneity within the development set, we implemented a hierarchical screening protocol. First, adjusting for six immune subsets estimated via Houseman’s deconvolution (Houseman *et al.* 2012), we computed *partial correlations* ($r_{\text{partial}}$) to mitigate composition bias, retaining the top 5000 significant CpGs.

Subsequently, to capture non-linear predictive patterns, we employed a Mamba-based regressor with Integrated Gradients (IG) (Sundararajan *et al.* 2017) for feature attribution. Iterative evaluation of IG-ranked loci minimized predictive error at 2700 features (Fig. S2, available as supplementary data at *Bioinformatics* online), forming a distilled, data-driven candidate set for the subsequent fusion stage (details in Supplementary Note 1, available as supplementary data at *Bioinformatics* online).

#### 2.2.2 Prior-guided screening and fusion

To anchor our model in established aging biology, we leveraged the proteomic atlas by Argentieri *et al.* (2024) to curate a hierarchical library of *20 core* (ProtAge20) and *204 broad-spectrum* aging proteins. Utilizing UCSC hg19 annotations, we extracted CpGs localized within their proximal promoters (TSS −2000 to +500 bp). We then implemented a Mechanism-Anchored Residual Ensemble framework, retaining all CpGs linked to the ProtAge20 network, while filtering broad-spectrum loci through dual-evidence criteria (intersection of proteomic relevance with the data-driven candidate pool).

This hierarchical fusion yielded 198 CpGs: 121 ProtAge20 anchors and 17 dual-evidence sites. To capture additional aging-related signals beyond the prior, the remaining 60 sites were purely data-driven residuals, with their number (k=60) determined via elbow-point analysis to balance predictive performance and model complexity (Fig. S1C, available as supplementary data at *Bioinformatics* online).

### 2.3 Construction of co-methylation topology

To model epigenetic dependencies, the selected CpG sites were represented as a weighted undirected graph G=(V,E), where V denotes the set of |V|=198 sites. The edge set E was constructed by integrating three complementary modalities: statistical co-methylation (S), genomic spatial proximity (G), and functional regulatory context (F). To harmonize different scales across modalities, a geometric mean-based aggregation was used for edge weighting. For any node pair $\left(v_{i},v_{j}\right)$, the edge weight $W_{ij}$ is defined as:


(1) $$W_{ij}={\left(\prod_{m\in \Omega_{ij}}m_{ij}\right)}^{\frac{1}{\left|\Omega_{ij}\right|}}$$

Where $\Omega_{ij}\subseteq \{S,G,F\}$ denotes the set of available (non-zero) modalities for the pair.

The individual components are defined as follows: *Statistical Co-methylation (*$S_{ij}$*)*, computed as the absolute Pearson correlation coefficient ($\left|r_{ij}\right|$) across samples; *Spatial Proximity (*$G_{ij}$*)*, modeled using an exponential decay function of genomic distance; and *Functional Context (*$F_{ij}$*)*, which assigns hierarchical weights (0.4–1.0) based on CpG genomic annotations (Islands > Shores > Shelves > Open Sea) (Eckhardt *et al.* 2006, Irizarry *et al.* 2009).

To reduce redundancy, a *k*-Nearest Neighbors (*k*-NN) sparsification strategy (Von Luxburg 2007) was applied. For each node, only the top-*k* neighbors (k=5) with the highest weights $W_{ij}$ were retained, resulting in a sparse adjacency matrix A used as input to the Graph Transformer.

### 2.4 GT-Mamba architecture

**Figure 1:**
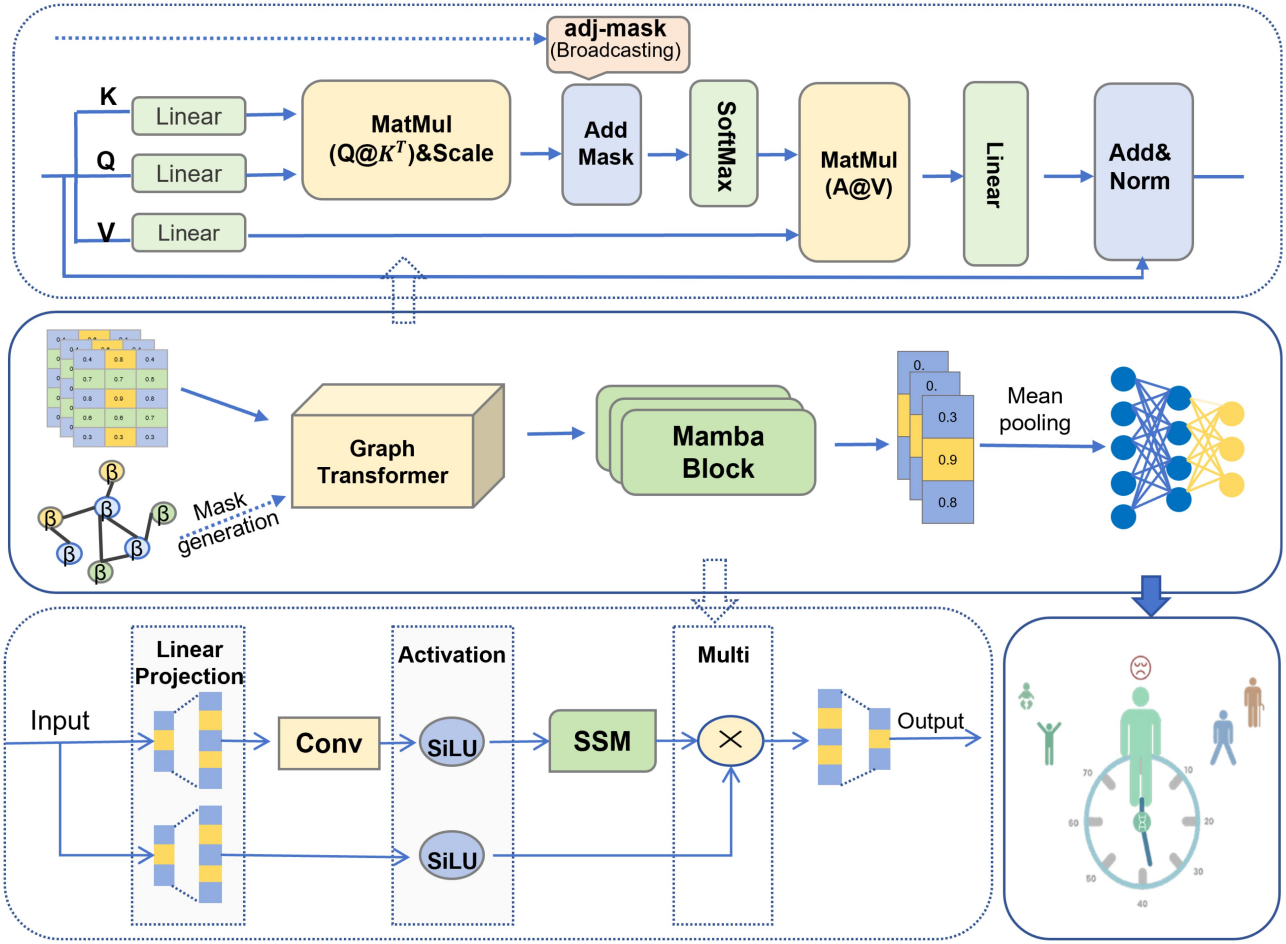
Overview of the GT-Mamba Architecture. The framework adopts a “Local Topology-Global Sequence” strategy. (Top) Structure-Aware Graph Transformer encodes local epigenetic contexts using the composite CpG adjacency matrix. (Middle) Data flows from methylation Beta values to the MLP predictor via global pooling. (Bottom) The Stacked Mamba Block captures distributed epigenetic interactions via the selective state space mechanism.

The *GT-Mamba* framework (Fig. 1) employs a hierarchical “Local Topology-Global Sequence” paradigm to estimate biological age.

#### 2.4.1 Local feature aggregation

A *Structure-Aware Graph Transformer* (Dwivedi and Bresson 2020) leverages the constructed composite epigenetic adjacency matrix to encode local contexts. This graph-based message passing ensures topology-preserving feature propagation across the multi-modal CpG network.

#### 2.4.2 Global context modeling

To integrate systemic signals across the selected CpG loci, the aggregated features are processed by a *Stacked Mamba Module* (Gu *et al.* 2021). Treating the CpG features as a trajectory discretized via Zero-Order Hold (ZOH), the state dynamics are defined as:


(2) $$h_{t}={\bar{A}}_{t}h_{t-1}+{\bar{B}}_{t}x_{t},\;y_{t}=C_{t}h_{t}$$

Crucially, these transition parameters are input-dependent, enabling the Selective State Space Model (SSM) to adaptively integrate information across CpG loci. This formulation supports the modeling of distributed and heterogeneous methylation signals, including dependencies among dispersed genomic sites.

#### 2.4.3 Prediction head

Finally, Global Average Pooling (GAP) synthesizes a sample-level representation from the output trajectory, followed by a multi-layer perceptron (MLP) to regress the final biological age.

### 2.5 Experimental design

Data from 15 cohorts were stratified into a development pool (10 cohorts; split 7:2:1) and an independent external test set (5 cohorts, including the large-scale EPIC 850k cohort GSE132203). We benchmarked against canonical clocks: *Horvath*, *Hannum*, and *PhenoAge* (Levine *et al.* 2018), as well as modern baselines such as *AltumAge* and *PC-Clocks*. To rigorously evaluate out-of-the-box generalization across platforms, all models were assessed using their raw predictions without any dataset-specific post-hoc linear bias correction.

For evaluation on the EPIC 850k array, 177 of the 198 GT-Mamba loci were directly mapped. To maintain input dimensionality, missing values were imputed using a *within-sample mean strategy*. For baseline models requiring fixed high-dimensional CpG inputs (e.g. AltumAge and PC-Clocks), standardized cohort-level mean imputation was applied, with zero-padding used for probes entirely absent from the EPIC platform, thereby enabling a consistent cross-platform evaluation.

The framework was implemented in PyTorch (Paszke *et al.* 2019) and optimized via AdamW (Loshchilov and Hutter 2017) with Cosine Annealing. Detailed hyperparameter settings and sensitivity analysis for the neighbor size *k* are listed in Table S2 and Supplementary Note 2, available as supplementary data at *Bioinformatics* online. Crucially, for the ablation study conducted on GSE132203, we enforced a strict constraint where variants (*GT-only*, *Mamba-only*) were *re-trained and evaluated* using the *identical set of 198 CpGs*, ensuring that performance gains are attributable solely to architectural components rather than feature selection bias.

Biological interpretation utilized Gene Ontology (GO) enrichment analysis via clusterProfiler (Wu *et al.* 2021). Significant biological processes (P<0.05) associated with the identified CpG sites were visualized to decode the underlying regulatory logic.

## 3 Results

### 3.1 Optimized graph topology balances parsimony and predictivity

By integrating data-driven statistical signals with protein-associated biological priors, an optimized feature topology was consolidated. The initial candidate pool was refined into a compact graph structure of 198 nodes (detailed list provided in Table S3, available as supplementary data at *Bioinformatics* online). This final set balances parsimony and predictivity, comprising 138 core anchor sites (linked to Core Aging Proteins) and 60 high-correlation residual supplements. On the *internal hold-out test set*, GT-Mamba achieved exceptional precision with an MAE of 2.85 years (Fig. S3, available as supplementary data at *Bioinformatics* online).

**Figure 2:**
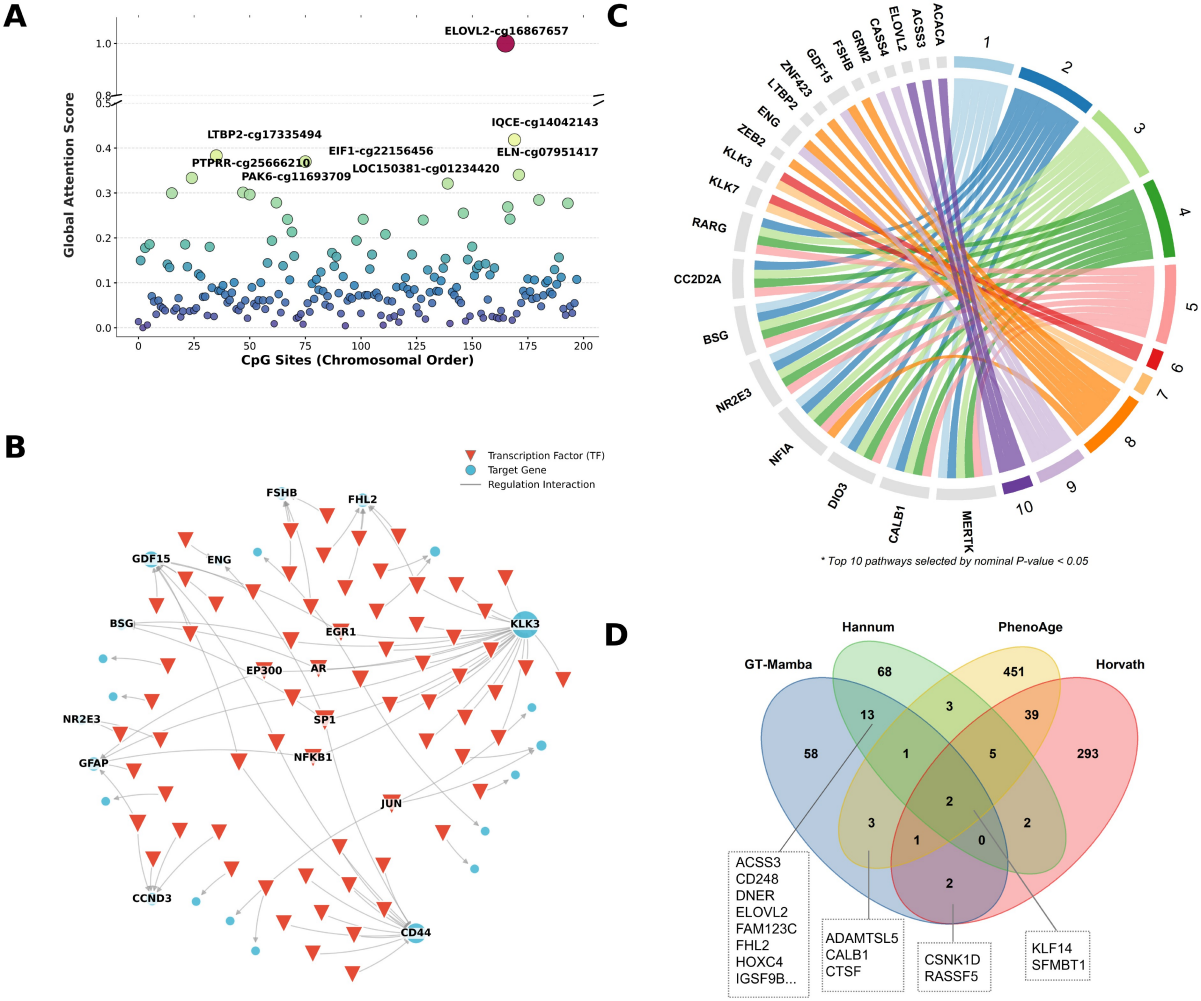
Identification of Key Regulatory Mechanisms and Functional Enrichment. (A) Global attention landscape visualizing genomic feature weights of the top identified CpG sites. (B) Transcription Factor (TF) regulatory network reconstructed via TRRUSTv2 (Blue nodes: TFs; Orange nodes: Target genes), revealing an SP1/AR-centric hierarchy. (C) Chord diagram of top-10 enriched GO Biological Processes (Nominal P<0.05). Key pathways include: (1) retina development; (6) antimicrobial peptide production; (8) TGF-β receptor signaling. (D) Venn diagram illustrating the intersection of feature genes between GT-Mamba and traditional clocks. The substantial unique fraction suggests the model’s ability to capture distinct, potentially non-linear, aging-associated molecular signatures.

Topological analysis reveals that the constructed co-methylation network contains 673 edges with a density of 3.45%, effectively mitigating over-smoothing risks. Conversely, the high average clustering coefficient (0.25) characterizes a “globally sparse, locally dense” architecture, providing the structural foundation for capturing high-order interactions within latent functional units. The biological validity of this structure is corroborated by the model’s global attention landscape (Fig. 2A), where GT-Mamba successfully pinpointed *salient nodes* like *ELOVL2* (cg16867657) (Garagnani *et al.* 2012) and centrality hubs like *ELN* (Small *et al.* 2011). Notably, certain loci (e.g. *ELOVL2*) were retained as prior-informed anchors during feature selection; therefore, their prominence in the attention landscape reflects both established biological relevance and the imposed prior structure, rather than a purely de novo discovery. Crucially, these nodes exhibit significant functional synergy rather than stochastic distribution. Functional enrichment analysis (Fig. 2C) confirms that these features are densely mapped to specific developmental pathways, suggesting that the model has captured specific biological program modules underlying aging (Chen *et al.* 2020).

### 3.2 Robust generalization capability across heterogeneous cohorts

To rigorously assess generalization, all models were evaluated in a strict out-of-the-box manner without any dataset-specific parameter fitting (e.g. post-hoc linear calibration). Under this setting, GT-Mamba was tested on five external cohorts (*N* = 1892), including a large-scale EPIC 850k dataset (GSE132203, *N* = 795).

Using raw, uncalibrated predictions (Fig. S5, available as supplementary data at *Bioinformatics* online), GT-Mamba achieved the lowest weighted Average MAE (4.43 years) across all cohorts (Table 1). It generalized well to the EPIC 850k array (MAE 4.94 years), despite differences in probe coverage. By utilizing the aforementioned within-sample strategy, GT-Mamba avoids cross-sample information leakage. Baseline models requiring fixed high-dimensional inputs (e.g. AltumAge and PC-Clocks) were additionally evaluated using standardized cohort-level imputation for missing EPIC probes. Under this consistent cross-platform setting, GT-Mamba consistently outperformed these baselines, further highlighting its superior out-of-the-box robustness.

**Table 1:** Performance benchmarking (Raw MAE in years).

Dataset	N	**Canonical Clocks**			Modern	**PC-Clocks**		**Ablation Study**		GT-Mamba
		Horv.	Hann.	Pheno.	Altum.	PC-Horv.	PC-Hann.	GT-only	Mamba-only	(Ours)
GSE40279	656	4.774	5.512	9.042	*3.473*	6.271	5.598	8.770	4.454	4.274
GSE61496	310	*4.300*	6.227	8.813	5.869	5.668	7.657	8.306	**2.705**	2.762
GSE72777	46	*2.278*	8.514	11.109	4.783	5.224	12.314	10.941	6.596	2.937
GSE77445	85	*7.640*	15.768	16.495	11.791	8.923	13.679	9.283	9.343	7.834
GSE132203	795	9.892	*6.165*	8.253	12.137	6.442	8.667	9.379	7.478	**4.940**
Weighted AvgMAE	–	6.914	6.436	9.047	7.891	*6.338*	7.750	9.026	5.710	**4.434**

GT-Mamba is compared against canonical clocks, modern deep learning models (AltumAge), and reliability-optimized PC-Clocks. The evaluation includes four 450k cohorts and one EPIC 850k dataset (GSE132203). All reported values represent raw MAE without post-hoc linear calibration to enable a standardized evaluation of out-of-the-box generalization. For the EPIC platform, missing probes in baseline models were imputed via cohort-level mean and zero-padding to enable a consistent evaluation. Bold: best overall; *Italic*: best baseline. Weighted Average MAE (AvgMAE) is computed based on the total sample size across external cohorts (*N* = 1892).

For comprehensive evaluation, we additionally compared GT-Mamba with healthspan and pace-of-aging models (GrimAgeV2, DunedinPACE, PhenoAge). As these models are not strictly optimized for chronological age regression, we report age acceleration correlations (calculated as linear regression residuals) and statistical significance (Supplementary Tables S5–S6 and Fig. S6, available as supplementary data at *Bioinformatics* online) rather than MAE.

Under a homologous distribution shift restricted to young individuals (GSE72777, age <35; Culig and Santer 2014), GT-Mamba maintained high precision (MAE 2.94 years), indicating robust generalization beyond the training distribution. Ablation studies across all external cohorts further validate the architecture. Both the Mamba-only (MAE 5.71 years) and GT-only (MAE 9.03 years) variants showed substantial performance degradation compared to the full model (MAE 4.43 years). The particularly sharp decline observed in the GT-only variant underscores that local co-methylation patterns alone are insufficient, highlighting the indispensable role of the Mamba architecture in integrating global contextual dependencies.

Biological specificity analysis across 450k and EPIC cohorts revealed negligible correlation between predicted age acceleration and estimated immune cell subsets (|r|<0.1; Fig. S4, available as supplementary data at *Bioinformatics* online), suggesting robustness against cell-type confounding across platforms.

### 3.3 Deciphering the systemic biological logic of epigenetic aging

To elucidate the biological signals underlying GT-Mamba’s predictions, we integrated functional enrichment, attention topology, and transcriptional regulatory network analysis into a unified multidimensional framework.

#### 3.3.1 Macroscopic functional architecture

The functional chord diagram (Fig. 2C; nominal P<0.05) reveals a landscape characterized by developmental processes and stromal remodeling. Specifically, the enrichment of “retina development” and “visual system development” is consistent with reported dysregulation of lineage-associated genes such as *NFIA* and *NR2E3* (Chen *et al.* 2020), potentially reflecting epigenetic drift within developmental programs. Furthermore, the diagram highlights functional associations among “TGF-beta receptor signaling,” “antimicrobial peptide production,” and “extracellular matrix organization,” suggesting coordinated biological processes relevant to aging.

#### 3.3.2 Microscopic core entities

To link these pathways to specific loci, model-derived attention weights (Fig. 2A) identified the top-weighted features. *ELOVL2* shows the highest attention weight, consistent with its established role as a robust epigenetic aging marker (Chen *et al.* 2020). In addition, *LTBP2* and *ELN* (Elastin)—both involved in extracellular matrix organization—rank among the top features, supporting the relevance of structural remodeling processes. Other high-weight features, including signaling regulators (*PTPRR*, *PAK6*) and cytoskeletal components (*IQCE*), further suggest that the model captures molecular patterns associated with cellular homeostasis and tissue integrity.

#### 3.3.3 Regulatory network context

Using the TRRUSTv2 database (Han *et al.* 2018), we reconstructed an upstream transcriptional regulatory network (Fig. 2B). The resulting network highlights *SP1* as a central regulator, coordinating with factors such as *JUN*, *NFKB1*, and *RELA*. In addition, the Androgen Receptor (*AR*) and its co-activator *EP300* are also identified as highly connected nodes, suggesting a potential role of endocrine-related signaling in the inferred network (Culig and Santer 2014). Downstream, genes such as *KLK3*, *CD44*, and *GDF15* appear as frequently connected targets. Collectively, these observations provide a systems-level view of regulatory relationships associated with the CpG features identified by the model, rather than implying a strictly causal hierarchy (Basisty *et al.* 2020).

### 3.4 Uncovering non-linear kinetics undetected by linear models

To delineate the distinct predictive landscape of GT-Mamba, the 198 identified core features were mapped to cognate genes and compared with established baseline clocks (Horvath, Hannum, and PhenoAge). As illustrated in Fig. 2D, GT-Mamba recapitulates canonical aging-associated loci shared with first-generation clocks, including *ELOVL2* and *FHL2*, while also identifying *KLF14* and *SFMBT1* as commonly selected features across models (Table S4, available as supplementary data at *Bioinformatics* online). In addition, the model shows overlap with the second-generation PhenoAge in genes associated with inflammatory processes (e.g. *GDF15*, *CD44*) and homeostatic regulation (*ADAMTSL5*), suggesting that it captures signals beyond chronological age prediction.

Notably, approximately 80% of the identified genes (e.g. *KLK3*, *NR2E3*, *GFAP*) are unique to GT-Mamba. These features, which are less frequently selected by linear models, are enriched in processes related to the tissue microenvironment and structural remodeling. This observation suggests that the proposed architecture is capable of capturing non-linear patterns within epigenetic data, thereby providing complementary molecular insights into aging-associated biological variation.

## 4 Discussion

### 4.1 The synergy of local topology and global context

This study addresses the trade-off between predictive accuracy and biological interpretability in epigenetic clock research. By engineering GT-Mamba—a hybrid framework synergizing Graph Transformers with State Space Models—we enable the modeling of complex non-linear aging dynamics while preserving biologically meaningful structure. A defining advantage of this architecture is its robustness against technical heterogeneity. Unlike traditional linear baselines (e.g. Horvath (Horvath 2013), Hannum (Hannum *et al.* 2013)) that often exhibit performance degradation under distribution shifts, GT-Mamba demonstrates consistent generalization (weighted Avg MAE 4.43 years on external validation) across independent datasets, including the challenging young cohort GSE72777. As evidenced by the ablation study, the graph topology acts as a structural regularizer, while the Mamba module captures distributed epigenetic interactions across CpG features; removing either component leads to noticeable performance degradation. This indicates that the topology-aware design enhances representation learning and helps distinguish biologically relevant aging signals from technical variation. Consequently, the proposed architecture reduces reliance on aggressive data harmonization and provides a practical framework for reliable *single-sample prediction* in heterogeneous settings.

### 4.2 Mechanistic insights: the “dual-track drive hypothesis” of systemic aging

Mechanistic interrogation of the 198 core loci suggests a “dual capture” pattern, reflecting both *developmental arrest features* (e.g. *NFIA*, *NR2E3*) and *functional decline markers* (e.g. *GDF15*, *MERTK*). Based on this observed duality, we outline the *“Dual-track Drive Hypothesis”* to delineate the evolution of systemic aging:

#### 4.2.1 Phase I: biophysical initiation

Consistent with established biological priors, our model assigned high importance to the downregulation of *ELOVL2*. Because *ELOVL2* is mandatorily retained as a prior-informed anchor in our framework, its prominent ranking validates the model’s alignment with known biology rather than representing a *de novo* discovery. As a regulator of LC-PUFA synthesis (Garagnani *et al.* 2012), its decline compromises membrane fluidity, mirroring retinal aging phenotypes (Chen *et al.* 2020). This observation aligns with the “retina development” enrichment identified in the model (*NR2E3*), suggesting that cross-tissue membrane rheological alterations are reflected in the learned representation. Compounded by *MERTK*-mediated efferocytosis impairment (Lemke 2019), these factors may represent the fundamental biophysical drivers of cellular decline.

#### 4.2.2 Phase II: antagonistic propulsion

The *AR*/*FHL2* axis is suggested as a potential strategy consistent with *Antagonistic Pleiotropy* (Williams 2001). This interpretation is consistent with evidence indicating that SP1 regulates *AR* expression (Hay *et al.* 2015, Yuan *et al.* 2005). In addition, chromatin accessibility analyses position *AR* within complex multi-factor regulatory programs (Tewari *et al.* 2012), supporting the biological plausibility of this inferred relationship. Under metabolic stress, cellular machinery may pivot to sustain sterile inflammation (via *KLK3*, *NFKB1*), a trade-off potentially associated with systemic wear.

#### 4.2.3 Phase III: entropic culmination

Ultimately, *GDF15*-driven microenvironmental fibrosis (Basisty *et al.* 2020), coupled with the transcriptional leakage of non-blood-specific genes (e.g. *NR2E3*) (Martinez-Jimenez *et al.* 2017), is associated with the collapse of epigenetic barriers maintaining cell identity. This process is consistent with a systemic escalation of disorder, aligning with the *Information Loss Theory of Aging* (Yang *et al.* 2023), and may mark the transition from regulated development to stochastic dysregulation.

### 4.3 Limitations and future perspectives

Despite the strong performance of GT-Mamba, several limitations should be noted.

A key limitation is that the mechanistic interpretations, including the “Dual-track Drive Hypothesis” and the SP1/AR-associated axis, are derived from computational modeling and should be regarded as hypothesis-generating rather than causal evidence. Experimental validation is required to confirm these relationships.

The topological pruning strategy, while improving efficiency, reduces the granularity of the original methylation landscape compared to dense array representations. Moreover, since the model is trained primarily on whole-blood data, its cross-tissue generalization remains to be systematically evaluated.

In addition, the hierarchical feature selection incorporates prior biological knowledge, including the mandatory retention of CpG sites associated with ProtAge20 proteins. While this enhances biological grounding and stability, it also introduces a degree of prior-guided bias. Accordingly, model interpretability reflects both prior-informed and data-driven features, rather than an unbiased identification of novel biomarkers.

Future work will focus on three directions: (1) *architectural extension* to multi-tissue and disease-specific settings; (2) *causal validation* of key regulatory hypotheses (e.g. *MERTK*, *GDF15*); and (3) *scaling up* to Whole-Genome Bisulfite Sequencing (WGBS) or integrating with emerging epigenetic foundation models (e.g. CpGPT, MethylGPT) to capture a more comprehensive epigenetic landscape.

## Supplementary Material

btag401_Supplementary_Data

## Data Availability

The source code and pre-trained models of GT-Mamba are publicly available at https://github.com/NENUBioCompute/GT-Mamba and archived on Zenodo (https://doi.org/10.5281/zenodo.19703155). All DNA methylation datasets used in this study are publicly available from the Gene Expression Omnibus (GEO), with detailed information provided in Table S1, available as supplementary data at *Bioinformatics* online. The proteomic aging atlas data were obtained from Argentieri *et al.* (2024).
