# Supplementary material for: GT-Mamba: a Topology-Aware Graph-State space model for robust and interpretable epigenetic age prediction
Source: Bioinformatics. 2026 Jun 16;42(7):btag401. doi: 10.1093/bioinformatics/btag401 (PMC13394493; doi:10.1093/bioinformatics/btag401)
Supplement: btag401_Supplementary_Data [file btag401_supplementary_data.zip › Supplementary Material.pdf]

Supplementary Material

Title: GT-Mamba: A Topology-Aware Epigenetic Clock Integrating Graph Transformer and Mamba for Interpretable Age Prediction

Han Wang[1,2,†], Hui Wang[1,†], Yanting Tong[1,‡], Yuanyuan Liu[1,‡], Qu Jing[4], Guan Ning Lin[5,*], and Li Zhang[3,*]

[1] School of Information Science and Technology, Institute of Computational Biology, Northeast Normal University, Changchun 130117, China

[2] School of Biomedical Engineering Excellence, China Pharmaceutical University, Nanjing 211198, China

[3] College of Computer Science and Engineering, Changchun University of Technology, Changchun 130051, China

[4] College of Computer Science and Technology, Jilin University, Changchun 130012, China

[5] Shanghai Mental Health Center, Shanghai Jiao Tong University School of Medicine; School of Biomedical Engineering, Shanghai Jiao Tong University, Shanghai, China

[†] Han Wang and Hui Wang contributed equally as co-first authors.

[‡] Yanting Tong and Yuanyuan Liu contributed equally to this work.

[*] Correspondence:

Guan Ning Lin, Shanghai Mental Health Center, Shanghai Jiao Tong University School of Medicine, Shanghai, China. E-mail: nickgnlin@sjtu.edu.cn

Li Zhang, College of Computer Science and Engineering, Changchun University of Technology, Changchun, Jilin 130051, China. E-mail: lizhang@ccut.edu.cn

Section	Title / Description	Page
**Supplementary Note 1**	**Technical Implementation of the Dual-Driven Feature Selection Strategy**<br>**Detailed mathematical formulation of the de-confounded partial correlation, Integrated Gradients (IG) settings, and the biological logic behind the stepwise feature expansion.**	**S-5**
**Supplementary Note 2**	**GT-Mamba Architecture and Implementation Details**<br>**Specifics of the dynamic graph construction (including k-NN sensitivity analysis), Mamba-based feature encoding mechanisms, and comprehensive training protocols.**	**S-8**
**Figure S1**	**Validation of the stepwise feature expansion and optimization strategy**<br>**Scatter plots and optimization curves illustrating the**	**S-9**

Section	Title / Description	Page
	performance evolution: from the biological anchor (20 sites) to the broad network (204 sites), and the final data-driven optimization (198 sites).	
Figure S2	Figure S2. Optimization and performance evaluation of the data-driven candidate pool. Stacked line plots showing the predictive performance—expressed as R-squared (R^2, top) and Mean Absolute Error (MAE, bottom)—across a range of feature subset sizes ($k = 500$ to $k = 3000$, step = 100) ranked by Mamba-based Integrated Gradients (IG). The vertical red dashed line indicates the optimal feature count ($k = 2{,}700$), representing the point of maximum predictive stability used for subsequent hierarchical fusion.	S-10
Figure S3	Performance evaluation on the internal test set<br>Scatter plots showing the prediction accuracy of GT-Mamba on the internal hold-out test set, demonstrating high precision and low error.	S-11
Figure S4	Robustness analysis against cell type heterogeneity<br>Validation demonstrating that the model's predictions are not driven by confounding shifts in cell type composition.	S-12
Figure S5	Generalization performance on independent external validation cohorts<br>Comprehensive evaluation on four independent datasets (GSE40279, GSE61496, GSE72777, GSE77445), demonstrating state-of-the-art generalization capability.	S-14
Figure S6	Prognostic validation and functional decoupling of GT-Mamba<br>Scatter plots illustrating linear correlations between GT-Mamba age acceleration and established second- and third-generation epigenetic clocks (PhenoAge, GrimAge2, and	S-16

Section	Title / Description	Page
	DunedinPACE).	
Figure S7	Functional enrichment analysis of the selected features<br>Gene Ontology (GO) dot plot of the 60 data-driven CpG sites, illustrating significantly enriched biological processes (e.g., embryonic organ development and skeletal system morphogenesis). The dot size represents gene count, and the color gradient indicates the adjusted P-value.	S-18
Table S1	Summary of DNA methylation datasets used in this study.<br>Detailed information on sample size, tissue type, age range, and microarray platform for all training and external validation cohorts.	S-19
Table S2	Hyperparameter settings for the GT-Mamba model<br>List of optimal parameters for the graph attention network and Mamba architecture, including the neighbor size (k) selection.	S-20
Table S3	List of the final 198 CpG markers and associated genes<br>The complete list of selected features, including CpG ID, mapped gene, and chromosome location. *(Note: Full table available as an external Excel file)*	S-21
Table S4	Overlap analysis with existing epigenetic clocks<br>Comparison of the 198 GT-Mamba CpGs with markers from Horvath, Hannum, and PhenoAge clocks, highlighting novel vs. conserved aging signatures. *(Note: Full table available as an external Excel file)*	S-22
Table S5	Comprehensive Performance Benchmarking of Predictive Epigenetic Clocks<br>Detailed evaluation metrics comparing GT-Mamba with canonical linear models, modern deep learning architectures, and PC-clocks across five independent cohorts. *(Note: Full*	S-23

Section	Title / Description	Page
	table available as an external Excel file)	
Table S6	**Prognostic Alignment of GT-Mamba with Next-Generation Clocks**<br>**Pearson correlations and P-values demonstrating GT-Mamba's capacity to reflect physiological decay captured by PhenoAge, GrimAgeV2, and DunedinPACE.** ***(Note: Full table available as an external Excel file)***	S-24

Supplementary Note 1: Technical Implementation of the Dual-Driven Feature Selection Strategy

1.Data-Driven Screening: De-confounded Partial Correlation Framework

To rigorously isolate intrinsic aging signals from cellular heterogeneity, we implemented a hierarchical screening protocol based on residual-based statistical filtering.

- **Cellular Proportion Estimation (Houseman Deconvolution)** A reference methylation profile matrix M was constructed based on purified cell data , encompassing eight components (CD8+ T, CD4+ T, NK, B cells, Monocytes, Eosinophils, Granulocytes, and undefined fractions). For any whole-blood sample methylation vector $\mathbf{Y}$, the corresponding proportion vector $\mathbf{P}$ is derived by minimizing the following objective function (constrained non-negative least squares, NNLS):

$$\min_{\mathbf{P}} \| \mathbf{Y} - \mathbf{MP} \|_2^2 \text{s.t.} \sum_{j=1}^{8} P_j = 1, P_j \geq 0 \quad (1)$$

- **Residual-Based De-confounding** To mitigate the confounding effects of cellular heterogeneity and avoid multicollinearity arising from the constant-sum constraint, Neutrophils and undefined fractions were excluded. The remaining six immune subsets ($\mathbf{P}'$) (CD8+ T, CD4+ T, NK, B cells, Monocytes, Eosinophils) were retained for regression correction. Specifically, for each CpG site, we constructed linear models to isolate intrinsic signals:

$$\text{Age} = \beta_{\text{age}}^T \mathbf{P}' + \varepsilon_{\text{age}} \quad (2)$$

$$CpG_i = \boldsymbol{\beta}_{\text{meth}}^T \mathbf{P}' + \varepsilon_{\text{meth}} \quad (3)$$

where $\mathbf{P}'$ denotes the proportion vector of the six subsets. The residuals ε_{age} and $\varepsilon_{\text{meth}}$ represent the corrected age and methylation levels, respectively, independent of cellular composition.

- **Partial Correlation Metric.**The intrinsic association strength is quantified by the partial correlation coefficient (r_{partial}):

$$r_{\text{partial}} = \text{Corr}(\varepsilon_{\text{age}}, \varepsilon_{\text{meth}}) \tag{4}$$

Based on partial correlation significance (P_{val}), we prioritized the top 5,000 CpGs.

2. Deep Feature Attribution via Integrated Gradients (IG)

To capture non-linear interactions, we trained a Mamba-based regressor on the pre-screened features. Input data were standardized (Z-score normalization), such that the zero-vector baseline ($\mathbf{x}' = \mathbf{0}$) corresponds to the population mean. The attribution score for the j-th feature, $IG_j(\mathbf{x})$, was approximated using Riemann summation with $m = 50$ steps:

$$IG_j(\mathbf{x}) \approx (\mathbf{x}_j - \mathbf{x}_j') \times \frac{1}{m}\sum_{k=1}^{m} \frac{\partial F\left(\mathbf{x}' + \frac{k}{m}(\mathbf{x} - \mathbf{x}')\right)}{\partial \mathbf{x}_j} \tag{5}$$

Where F represents the Mamba model. The final global feature importance was determined by calculating the mean absolute attribution across all validation samples.

3. Prior-Guided Screening: Targeting CpGs Linked to Aging Proteins

To anchor the model in established aging physiology, we leveraged the landmark proteomic atlas by Argentieri et al. We constructed a hierarchical marker library stratified into two networks:

Core Network (ProtAge20): 20 Core Aging Proteins, prioritized through Mendelian Randomization as high-causality drivers.

Broad-Spectrum Network: 204 Broad-Spectrum Proteins, expanding the scope to capture systemic aging signals.

Using UCSC hg19 annotations, we extracted all CpG sites situated within the cis-regulatory window (TSS -2000 bp to +500 bp) of these target genes. As shown in Supplementary Figure S1A and S1B, expanding from the Core network (MAE = 6.38) to the Broad-Spectrum network (MAE = 4.64) significantly improved predictive accuracy, validating the necessity of the hierarchical expansion strategy.

4. Mechanism-Anchored Feature Assembly Protocol

To construct the final hybrid feature space, we implemented a three-tier assembly logic:

Tier 1 (Anchor): All CpG sites mapped to the 20 core ProtAge20 proteins were mandatorily retained to ensure biological grounding, yielding 121 ProtAge20 anchors.

Tier 2 (Filter): For the expanded pool of 204 broad-spectrum proteins, we applied a strict dual-evidence filter. By intersecting these prior-guided loci with the highly distilled 2,700-CpG candidate pool, we retained only the overlapping features, yielding 17 dual-evidence sites.

Tier 3 (Rescue): To capture latent aging mechanisms outside the established proteomic network, we recruited top-ranking purely data-driven residuals from the remaining loci within the 2,700-CpG pool.

Specifically, starting from the 138 biological anchors (121 Tier 1 and 17 Tier 2 sites), we sequentially evaluated the inclusion of Tier 3 candidates (step = 10). As demonstrated in Supplementary Figure S1C, an elbow-point analysis determined the optimal number of these residual features (k=60) to maximize predictive accuracy without overfitting. This process effectively balanced conserved biological drivers with high-precision empirical signals, yielding the final 198-CpG topology.

Supplementary Note 2: GT-Mamba Architecture and Implementation Details

1. Dynamic Graph Construction (Parameter Sensitivity)

To capture the non-Euclidean interactions among the selected CpG sites, we constructed a sparse graph $\mathcal{G}$ where connections were defined by a k-Nearest Neighbors (k-NN) strategy. While the general graph framework is introduced in the main text, the specific choice of neighborhood size k was determined through a rigorous sensitivity analysis ($k \in \{5,10,15,20\}$). Empirical results demonstrated that $k = 5$ yielded the best predictive performance (MAE = 2.8537), outperforming larger neighborhood settings ($k = 10$: MAE=2.9460; $k = 15$: MAE=2.9536; $k = 20$: MAE=2.8987). This confirms that a highly sparse topology ($k = 5$) effectively preserves local biological manifold structures while minimizing noise propagation, justifying our hyperparameter selection.

2. Mamba-based Feature Encoding Implementation

Following the continuous-time State Space Model (SSM) formulation detailed in the Main Text (Methods), we implemented the Mamba layer using the Zero-Order Hold (ZOH) discretization method. This step converts the continuous parameters $(\mathbf{A}, \mathbf{B})$ into discrete parameters $(\overline{\mathbf{A}}, \overline{\mathbf{B}})$ aligned with the sampling resolution of the input data. This implementation allows the model to efficiently process long sequences with linear complexity$O(L)$, ensuring scalability for high-dimensional epigenetic features.

3. **Training Strategy and Hyperparameters**

The model was implemented in PyTorch and optimized using the Mean Squared Error (MSE) loss function (as defined in the Main Text). The training process adhered to the following specific protocols: Optimizer: AdamW with an initial learning rate of $1 \times 10^{-4}$and weight decay of1×10^{-4}. Scheduler：A ReduceLROnPlateau scheduler was employed to halve the learning rate if the validation loss did not improve for 10 epochs.Early Stopping: Training was automatically terminated if the validation MAE showed no improvement for 30 consecutive epochs to prevent overfitting. Hardware: All experiments were conducted on a single NVIDIA GPU.

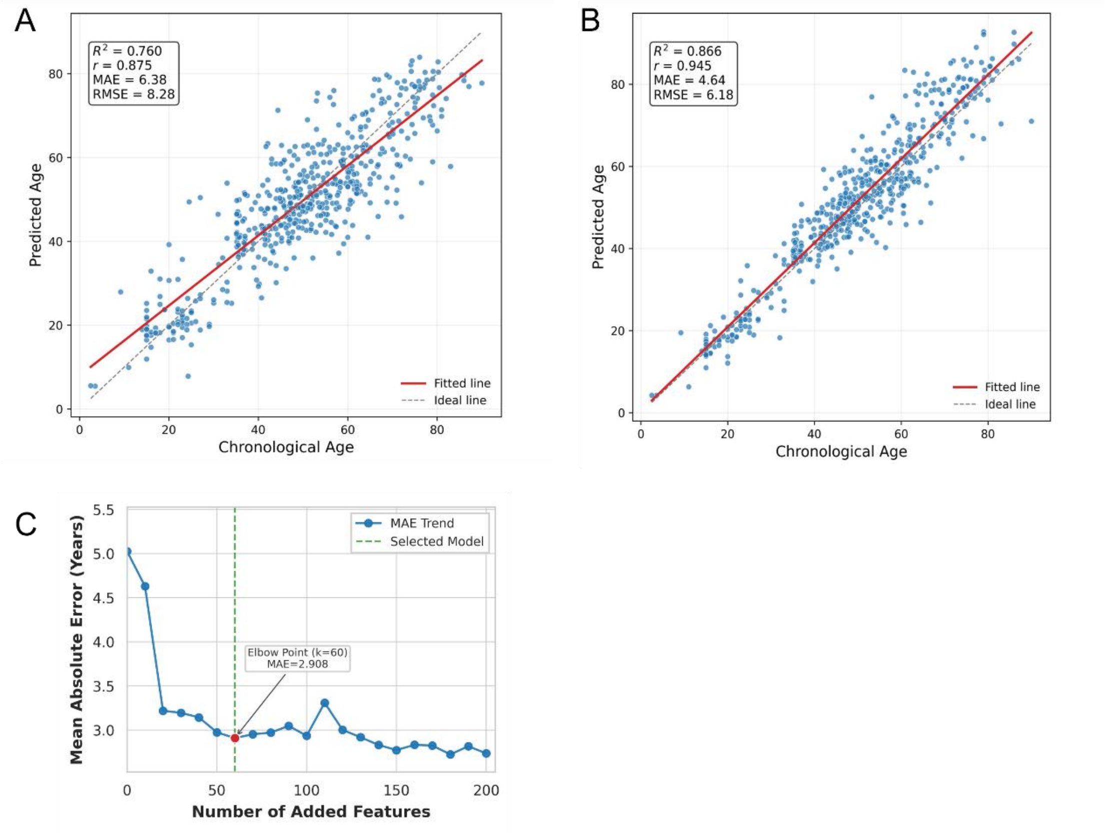

Supplementary Figure S1: Validation of the stepwise feature expansion and optimization strategy using the GT-Mamba architecture. (A) Performance baseline using 20 core aging proteins. Scatter plot showing the baseline prediction accuracy ($R^2 = 0.760$, MAE = 6.38 years) using only CpGs mapped to the 20 core drivers. **(B) Performance improvement with broad-spectrum network expansion.** Scatter plot showing the significant accuracy gain ($R^2 = 0.866$, MAE = 4.64 years) after incorporating CpGs mapped to the expanded network of 204 broad-spectrum aging proteins. **(C) Incremental optimization of the data-driven residual features.** The MAE trend curve illustrates the performance impact of the data-driven recruitment process. Starting from a fixed base set of prior-guided anchors, purely data-driven candidates were sequentially recruited. The trajectory reveals a distinct elbow point at$k = 60$ (MAE = 2.908 years), determining the optimal balance between maximizing predictive accuracy and preventing model overfitting, which ultimately yields the highly refined 198-CpG final topology.

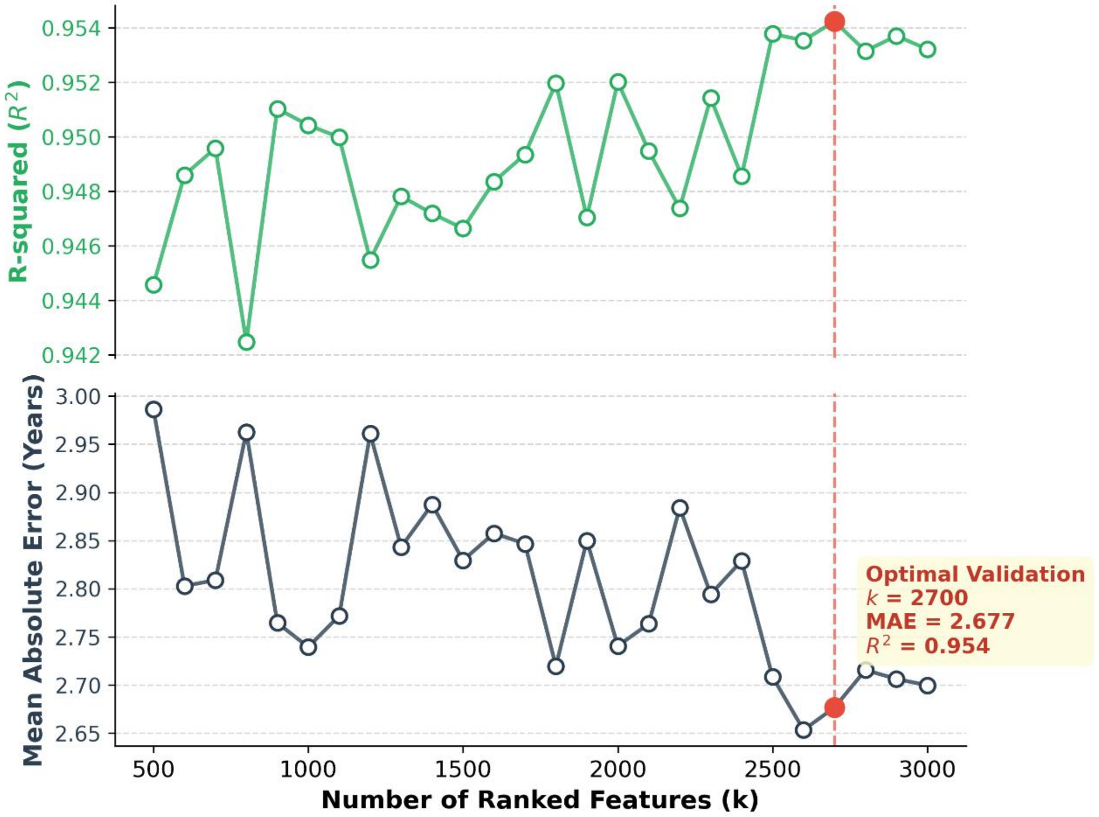

Supplementary Figure S2: Optimization of the purely data-driven candidate pool. The stacked line plots illustrate the validation performance—R-squared (top, green) and Mean Absolute Error (bottom, dark blue)—across different feature subset sizes (from 500 to 3,000 CpGs, step = 100) ranked by Integrated Gradients. The optimal predictive balance was achieved at $k = 2{,}700$ features (highlighted by the red dashed line, MAE = 2.677, R^2= 0.954), which was subsequently selected as the highly distilled candidate pool for the hierarchical fusion stage.

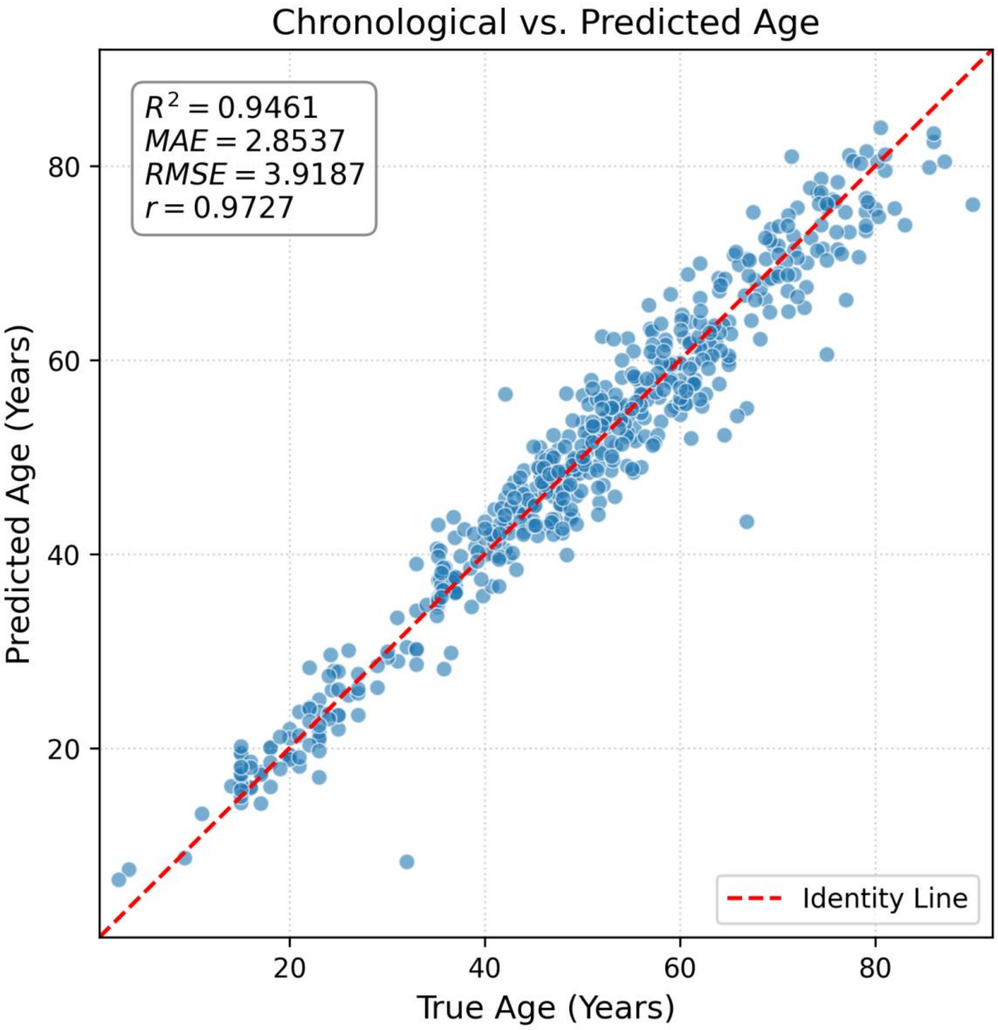

Supplementary Figure S3: Performance evaluation on the internal test set. Scatter plot depicting the agreement between true chronological age (x-axis) and epigenetic age predicted by the GT-Mamba model (y-axis) on the internal hold-out test set. The red dashed line represents the identity line , indicating perfect prediction. The exceptional accuracy and minimal error (MAE = 2.85 years) demonstrate the model's high precision and effective capture of aging patterns within the source population.

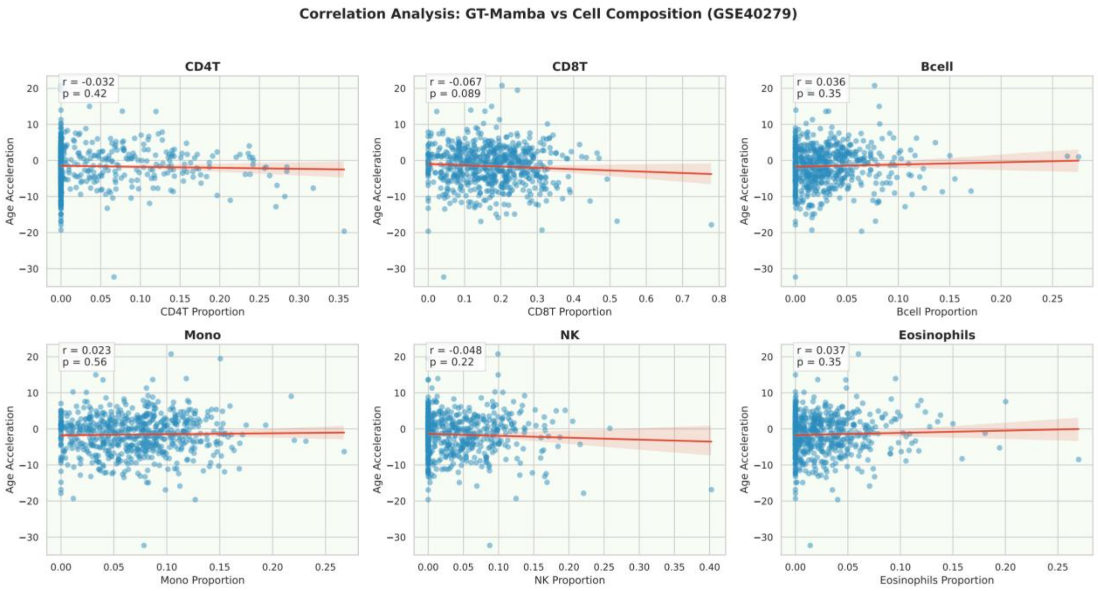
Correlation Analysis: GT-Mamba vs Cell Composition (GSE40279)
CD4T
r = -0.032
p = 0.42
CD4T Proportion
CD8T
r = -0.067
p = 0.089
CD8T Proportion
Bcell
r = 0.036
p = 0.35
Bcell Proportion
Mono
r = 0.023
p = 0.56
Mono Proportion
NK
r = -0.048
p = 0.22
NK Proportion
Eosinophils
r = 0.037
p = 0.35
Eosinophils Proportion
Age Acceleration

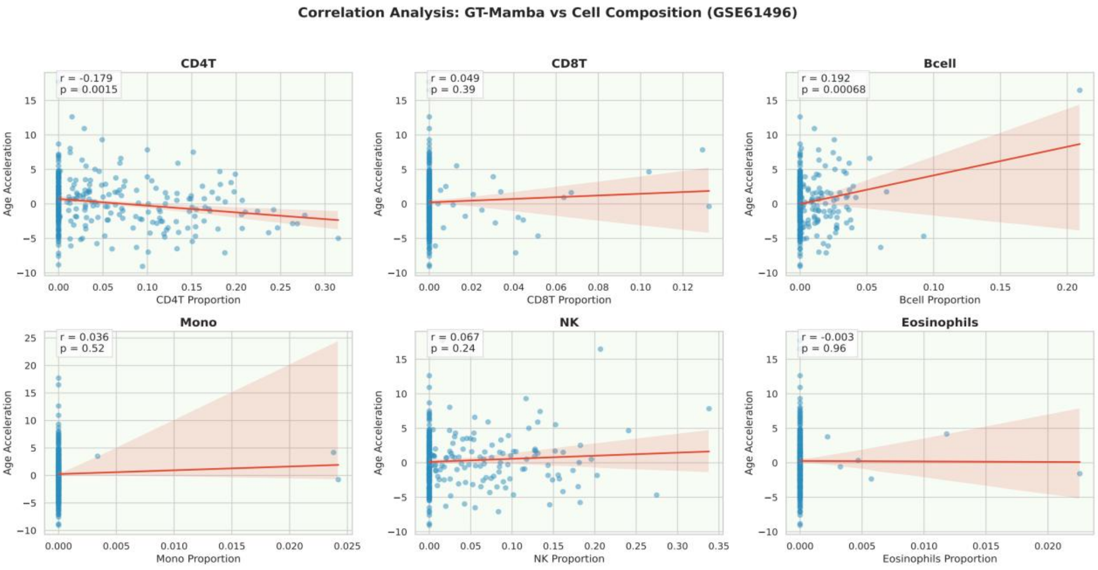
Correlation Analysis: GT-Mamba vs Cell Composition (GSE61496)
CD4T
r = -0.179
p = 0.0015
CD4T Proportion
CD8T
r = 0.049
p = 0.39
CD8T Proportion
Bcell
r = 0.192
p = 0.00068
Bcell Proportion
Mono
r = 0.036
p = 0.52
Mono Proportion
NK
r = 0.067
p = 0.24
NK Proportion
Eosinophils
r = -0.003
p = 0.96
Eosinophils Proportion
Age Acceleration

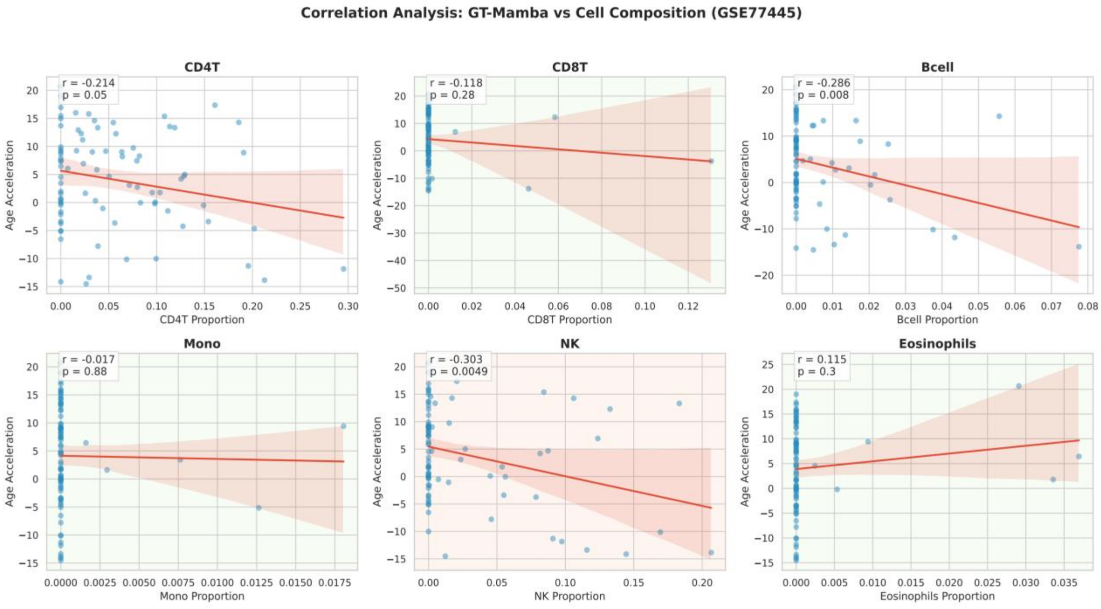
Correlation Analysis: GT-Mamba vs Cell Composition (GSE77445)
CD4T
r = -0.214
p = 0.05
CD4T Proportion
CD8T
r = -0.118
p = 0.28
CD8T Proportion
Bcell
r = -0.286
p = 0.008
Bcell Proportion
Mono
r = -0.017
p = 0.88
Mono Proportion
NK
r = -0.303
p = 0.0049
NK Proportion
Eosinophils
r = 0.115
p = 0.3
Eosinophils Proportion
Age Acceleration

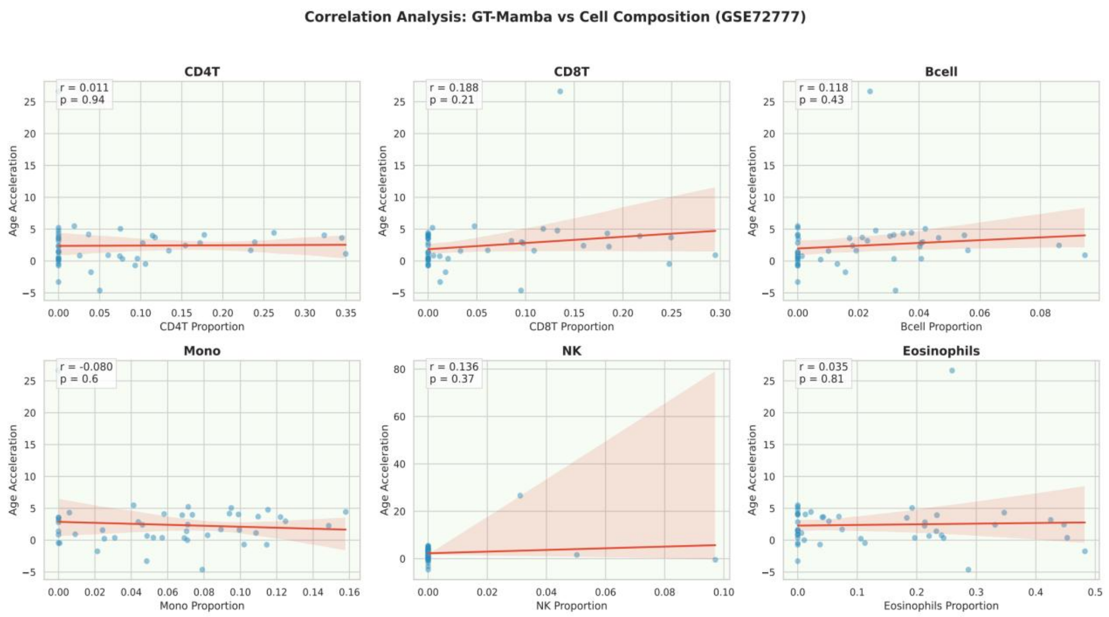

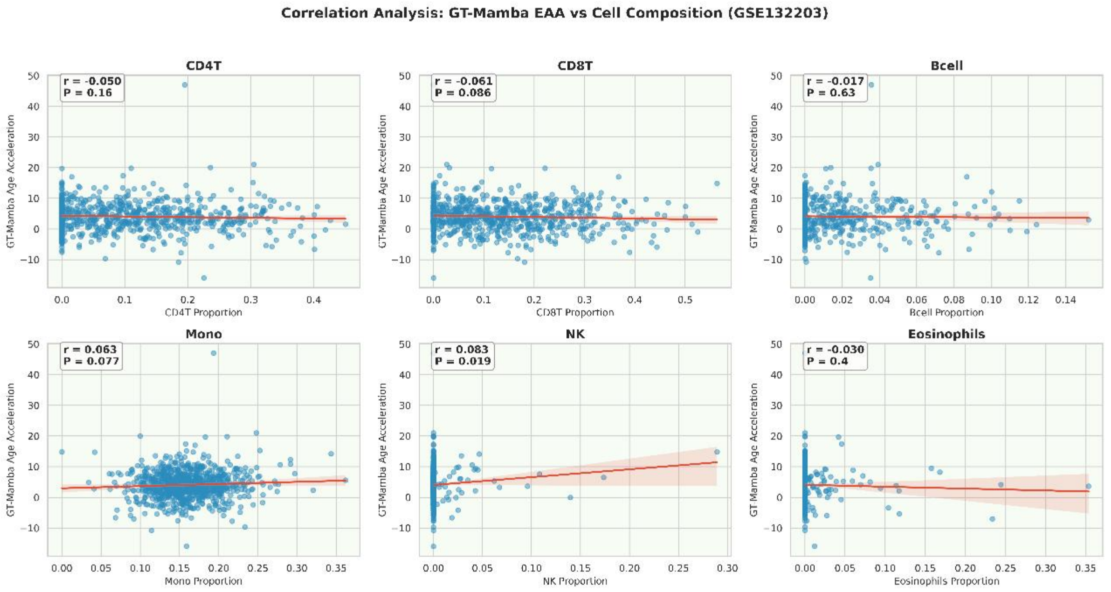

Supplementary Figure S4. Robustness of GT-Mamba against immune cell composition heterogeneity across multiple validation cohorts. Scatter plots illustrate the Pearson correlation analysis between GT-Mamba predicted Age Acceleration (EAA) and the proportions of six major immune cell types (CD4T, CD8T, B-cell, Monocytes, NK cells, and Eosinophils). This analysis was conducted across five independent external validation datasets, encompassing four 450k array cohorts (e.g., GSE40279) and a large-scale modern EPIC 850k array cohort (GSE132203).The correlation coefficient (r) and P-value (p) are annotated in each subplot. Notably, in the largest validation cohort (GSE132203, $N=795$), GT-Mamba predictions remained statistically independent of cell type proportions, with all

correlation coefficients $|r| < 0.1$. This consistency across both 450k and EPIC platforms demonstrates that the model successfully captures intrinsic, platform-independent epigenetic aging signals rather than confounding shifts in cellular composition.

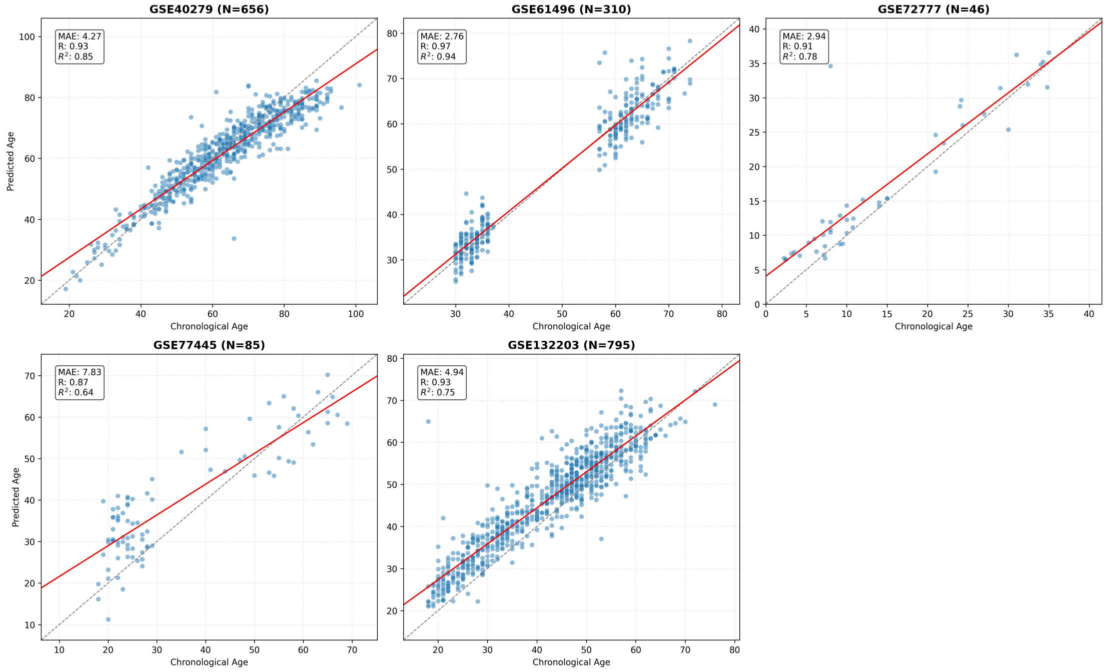

Supplementary Figure S5: Assessment of model generalization and cross-platform robustness on independent external cohorts. Scatter plots illustrate the chronological age (x-axis) versus the predicted epigenetic age (y-axis) by the GT-Mamba model across five independent validation datasets (GSE40279, GSE61496, GSE72777, GSE77445, and GSE132203). The red solid line represents the linear regression fit, while the gray dashed line denotes the identity line ($y = x$, indicating perfect prediction). Performance metrics, including Mean Absolute Error (MAE), Pearson correlation coefficient (R), and coefficient of determination (R^2), are provided in the upper-left box of each panel, with sample sizes (N) indicated in the subplot titles. The consistent, high-accuracy performance across these diverse cohorts—particularly on the EPIC 850k array dataset (GSE132203)—demonstrates the robust generalizability and cross-platform reliability of the proposed GT-Mamba architecture and the identified 198-CpG signature.

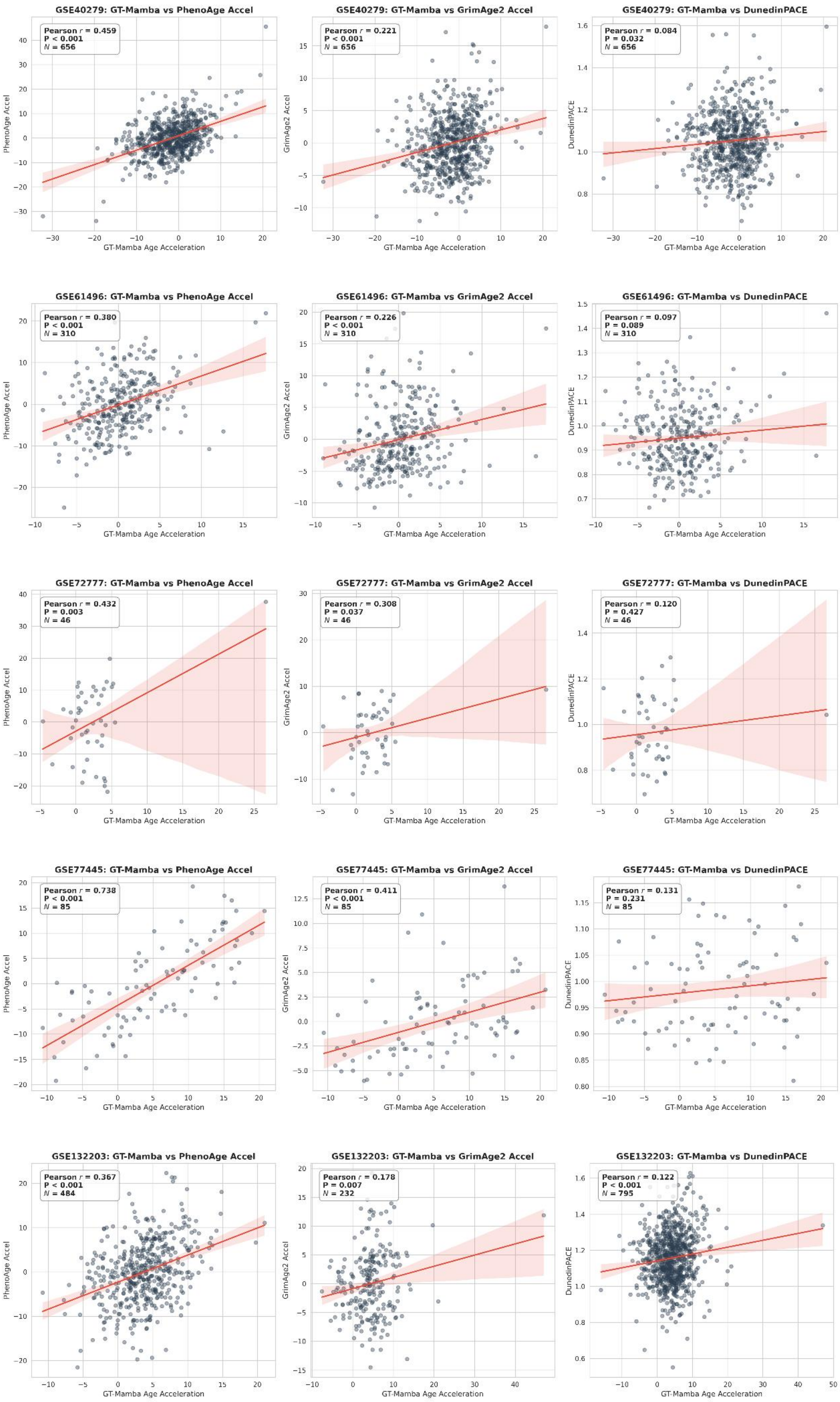

GSE40279: GT-Mamba vs PhenoAge Accel
Pearson r = 0.459
P < 0.001
N = 656
GSE40279: GT-Mamba vs GrimAge2 Accel
Pearson r = 0.221
P < 0.001
N = 656
GSE40279: GT-Mamba vs DunedinPACE
Pearson r = 0.084
P = 0.032
N = 656
GSE61496: GT-Mamba vs PhenoAge Accel
Pearson r = 0.380
P < 0.001
N = 310
GSE61496: GT-Mamba vs GrimAge2 Accel
Pearson r = 0.226
P < 0.001
N = 310
GSE61496: GT-Mamba vs DunedinPACE
Pearson r = 0.097
P = 0.089
N = 310
GSE72777: GT-Mamba vs PhenoAge Accel
Pearson r = 0.432
P = 0.003
N = 46
GSE72777: GT-Mamba vs GrimAge2 Accel
Pearson r = 0.308
P = 0.037
N = 46
GSE72777: GT-Mamba vs DunedinPACE
Pearson r = 0.120
P = 0.427
N = 46
GSE77445: GT-Mamba vs PhenoAge Accel
Pearson r = 0.738
P < 0.001
N = 85
GSE77445: GT-Mamba vs GrimAge2 Accel
Pearson r = 0.411
P < 0.001
N = 85
GSE77445: GT-Mamba vs DunedinPACE
Pearson r = 0.131
P = 0.231
N = 85
GSE132203: GT-Mamba vs PhenoAge Accel
Pearson r = 0.367
P < 0.001
N = 484
GSE132203: GT-Mamba vs GrimAge2 Accel
Pearson r = 0.178
P = 0.007
N = 232
GSE132203: GT-Mamba vs DunedinPACE
Pearson r = 0.122
P < 0.001
N = 795
PhenoAge Accel
GrimAge2 Accel
DunedinPACE
GT-Mamba Age Acceleration

Supplementary Figure S6. Prognostic validation and computational decoupling of GT-Mamba across independent cohorts. Scatter plots illustrate the linear correlations between the epigenetic age acceleration (AgeAccel) predicted by GT-Mamba and three established second- and third-generation epigenetic clocks (PhenoAge, GrimAge2, and DunedinPACE). To rigorously eliminate chronological baseline biases, all AgeAccel values were standardized as residuals from regressing predicted age onto chronological age.

The consistent, statistically significant correlations with PhenoAge (r: 0.36–0.73) validate GT-Mamba's robust capacity to capture systemic physiological wear-and-tear, highlighting the advantage of our hybrid feature extraction that anchors on biological priors before mining data-driven residuals. Consequently, by avoiding the localized lifestyle and immune confounders (e.g., smoking pack-years) heavily weighted in GrimAge2, GT-Mamba exhibits markedly weaker correlations with GrimAge2 ($r \approx 0.17 - 0.41$), effectively isolating fundamental epigenetic drift. Furthermore, the systematically near-zero correlations with DunedinPACE ($r \approx 0.1$) corroborate a distinct functional decoupling, confirming that GT-Mamba acts strictly as an orthogonal "state" estimator of cumulative biological aging.

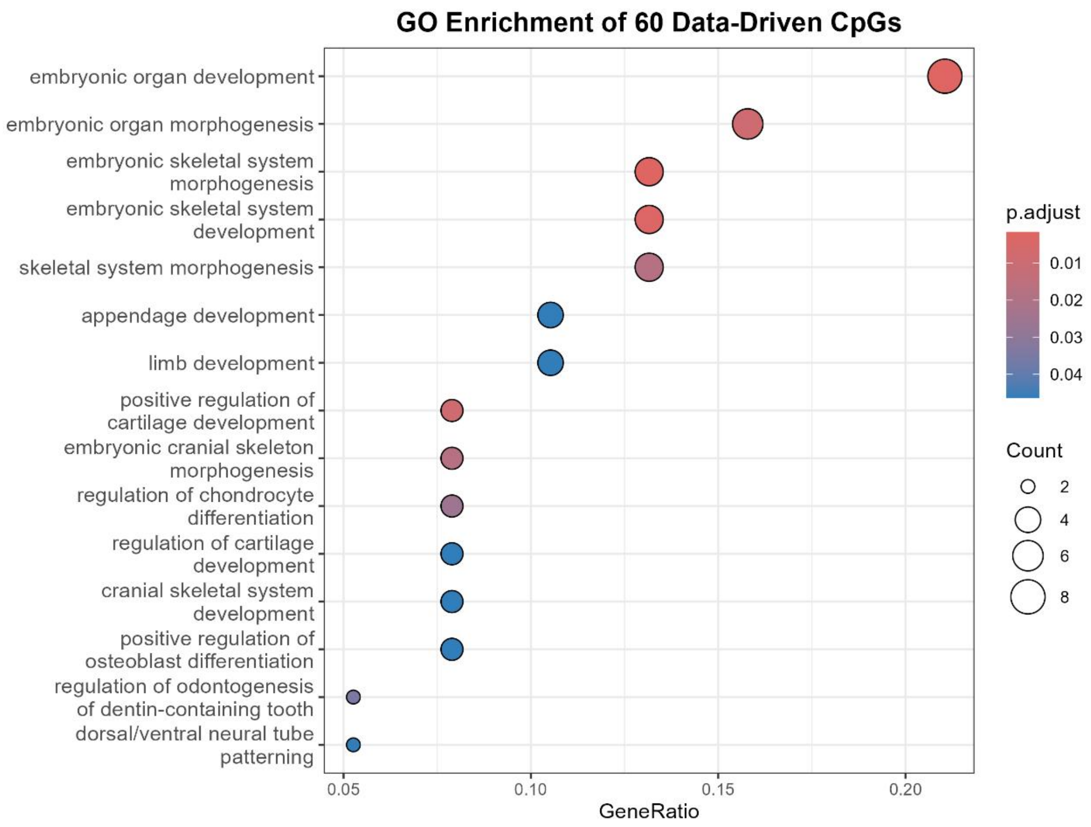

Supplementary Figure S7. Gene Ontology (GO) enrichment analysis of the 60 purely data-driven CpG sites. The dot plot illustrates the top enriched GO Biological Process terms associated with the 60 data-driven features (independent of proteomic priors) retained in the final GT-Mamba model. The x-axis represents the GeneRatio (the proportion of input genes mapped to a specific GO term), and the y-axis lists the enriched biological pathways. The size of each dot corresponds to the number of mapped genes (Count), while the color gradient indicates the statistical significance (adjusted P-value), ranging from highly significant (red) to less significant (blue). The results highlight a strong enrichment in developmental processes and tissue morphogenesis (e.g., embryonic organ development, skeletal system morphogenesis), suggesting that these purely data-driven epigenetic markers successfully capture fundamental developmental signatures underlying the aging process.

Supplementary Table S1. Demographic characteristics of the 15 public DNA methylation datasets integrated in this study.

Dataset ID	Sample Size (N)	Tissue	Platform	Age (Mean ± SD)	Age Range	Group
GSE50660	464	Whole Blood	Illumina 450k	55.4 ± 6.7	[38.0, 67.0]	Training
GSE53128	43	Whole Blood	Illumina 450k	62.2 ± 6.8	[47.3, 76.5]	Training
GSE55763	2711	Whole Blood	Illumina 450k	51.0 ± 10.1	[23.7, 75.0]	Training
GSE60132	192	Whole Blood	Illumina 450k	36.2 ± 18.8	[6.0, 85.0]	Training
GSE64495	113	Whole Blood	Illumina 450k	37.5 ± 18.7	[2.3, 73.7]	Training
GSE65638	16	Whole Blood	Illumina 450k	26.2 ± 4.4	[21.0, 32.0]	Training
GSE72773	310	Whole Blood	Illumina 450k	65.6 ± 13.9	[35.1, 91.9]	Training
GSE72775	335	Whole Blood	Illumina 450k	70.2 ± 10.3	[36.5, 90.5]	Training
GSE73103	355	Whole Blood	Illumina 450k	20.5 ± 4.8	[14.0, 34.0]	Training
GSE87571	727	Whole Blood	Illumina 450k	47.4 ± 21.0	[14.0, 94.0]	Training
GSE40279	656	Whole Blood	Illumina 450k	64.0 ± 14.7	[19.0, 101.0]	Validation
GSE61496	310	Whole Blood	Illumina 450k	48.5 ± 15.4	[30.0, 74.0]	Validation
GSE72777	46	Whole Blood	Illumina 450k	14.7 ± 10.4	[2.2, 35.0]	Validation
GSE77445	85	Whole Blood	Illumina 450k	33.8 ± 15.9	[18.0, 69.0]	Validation
GSE132203	795	Whole Blood	Illumina 850k	42.3 ±12.7	[18.0-76.0]	Validation

Note: Datasets were sourced from NCBI GEO. Samples from ten 450k datasets were randomly split (70% training, 20% validation, 10% testing). The remaining five datasets (including EPIC 850k GSE132203) were used strictly for independent external validation.

Supplementary Table S2. Detailed hyperparameter settings and model architecture configurations used in this study.

Category	Parameter	Value	Description
**Graph Construction**	**Neighbor Size (k)**	**5**[†]	Number of nearest neighbors for graph sparsification.
**General Training**	Optimizer	AdamW	Optimization algorithm for weight updates.
	Learning Rate	1×10^{-4}	Initial learning rate.
	Weight Decay	1×10^{-4}	L2 regularization factor to prevent overfitting.
	Batch Size	8	Number of samples per training batch.
	Max Epochs	400	Maximum number of training iterations.
	Early Stopping Patience	30	Epochs to wait for improvement before stopping.
	LR Scheduler	ReduceLROn Plateau	Factor=0.5, Patience=10.
**Graph Transformer**	Model Dimension (d_{model})	64	Dimension of input node features.
	Num Heads	4	Number of multi-head attention mechanisms.
	Attention Dropout	0.1	Dropout rate within the graph attention layer.
**Mamba (SSM)**	Mamba Layers	2	Number of stacked Selective State Space blocks.
	State Dimension (d_{state})	16	Expansion factor of the SSM state.
	Conv Width	4	Size of the local 1D convolution kernel.
**Prediction Head**	Hidden Dimension	32	Dimension of the regressor's hidden layer.
	Activation Function	GELU	Non-linear activation function.
	Head Dropout	0.1	Dropout rate applied before the final regression.

5[†] Note: The neighbor size was selected based on a sensitivity analysis, where k=5 achieved the lowest validation error (MAE=2.8537) compared to k=10, 15, 20.

Supplementary Table S3: List of the 198 CpG sites used as input features

Description: This table provides the exact list of the 198 DNA methylation markers (CpG sites) automatically selected by the GT-Mamba framework. These sites represent the core input features identified by the model to capture aging kinetics. Providing these unique identifiers ensures the reproducibility of the study, allowing researchers to map these sites to standard Illumina manifests for downstream analysis or validation in independent cohorts.

Data Structure (Sheet 1):

CpG ID: The unique Illumina probe identifier (e.g., cg05575921) corresponding to the selected methylation sites.

File Location:

File Name: Supplementary_Data.xlsx

Tab Name: Table S3_CpG_Markers

Supplementary Table S4: Comparative Gene Lists

Description: This table presents a gene-level intersection analysis comparing the biological features captured by GT-Mamba against three canonical epigenetic clocks: Horvath's multi-tissue clock (2013), Hannum's blood-based clock (2013), and PhenoAge (2018). The comparison highlights both the shared aging biomarkers (confirming biological validity) and the novel genes exclusively identified by the deep topology-aware paradigm of GT-Mamba (demonstrating new biological insights).

Data Structure (Sheet 2): The dataset is organized into four comparative columns for Venn diagram analysis:

GT-Mamba Genes: The list of 198 gene symbols mapped from the GT-Mamba CpG markers.

Horvath (2013) Genes: Genes associated with the 353 CpG sites from Horvath's clock.

Hannum (2013) Genes: Genes associated with the 71 CpG sites from Hannum's clock.

PhenoAge (2018) Genes: Genes associated with the 513 CpG sites from the PhenoAge clock.

File Location:

File Name: Supplementary_Data.xlsx

Tab Name: Table S4_Gene_Comparisons

Supplementary Table S5: Comprehensive Performance Benchmarking of Predictive Epigenetic Clocks

Description: This table presents the detailed evaluation metrics — Mean Absolute Error (MAE), Pearson correlation coefficient (R), and R-squared (R2) — for GT-Mamba compared against canonical linear models, modern deep learning architectures (AltumAge), and reliability-optimized PC-clocks across five independent cohorts. To ensure a rigorous and consistent benchmarking on the EPIC 850k platform (GSE132203), baseline models requiring fixed high-dimensional inputs were evaluated using a standardized cohort-level mean imputation and zero-padding strategy to resolve probe mismatch issues. Under this unified cross-platform setting, GT-Mamba consistently outperforms all baseline clocks in raw out-of-the-box prediction, highlighting its superior structural robustness against technical heterogeneity.

Data Structure:

Dataset: The GEO accession number of the independent cohorts used for external validation.

Samples (N): The total number of samples in each respective cohort.

Model Metrics: The MAE, Pearson R, and R2 values computed for each evaluated epigenetic clock (including Horvath, Hannum, PhenoAge, AltumAge, PC-Horvath, PC-Hannum, PC-PhenoAge, and GT-Mamba).

File Location:

File Name: Supplementary_Data.xlsx

Tab Name: Table S5_Benchmarking

Supplementary Table S6: Prognostic Alignment of GT-Mamba with Next-Generation Clocks

Description: This table details the Pearson correlation coefficients (R) and corresponding P-values between the chronological age predicted by GT-Mamba and the biological aging or prognostic signals captured by next-generation tools, including PhenoAge, GrimAgeV2, and the DunedinPACE rate of aging. The highly significant positive correlations provided here demonstrate GT-Mamba's robust capacity to capture physiological decay and biological pace, despite being trained solely on chronological time.

Data Structure:

Dataset: The GEO accession number of the independent cohorts.

Samples (N): The total number of samples in each respective cohort.

Correlation Metrics: The Pearson correlation coefficient (R) and statistical significance (P-value) calculated between the raw predictions of GT-Mamba and the respective prognostic clock outputs (PhenoAge, GrimAgeV2, and DunedinPACE).

File Location:

File Name: Supplementary_Data.xlsx

Tab Name: Table S6_Prognostic_Alignment
